# Bream: an open-source deep learning framework for simultaneous base calling and DNA methylation detection on novel nanopore sequencing platforms

**DOI:** 10.3389/fgene.2026.1743148

**Published:** 2026-01-14

**Authors:** Hui-Cong Yao, Bo Wu, Chen-Liang Ye, Xin Bai, He-Xu Chen, Geng Hu, Chuan-Le Xiao

**Affiliations:** 1 School of Artificial Intelligence, Sun Yat-sen University, Zhuhai, China; 2 State Key Laboratory of Ophthalmology, Zhongshan Ophthalmic Center, Sun Yat-sen University, Guangdong Provincial Key Laboratory of Ophthalmology and Visual Science, Guangzhou, China; 3 Bioinformatics and Product Development Department, Qitan Technology (Beijing) Co., Ltd, Beijing, China

**Keywords:** base calling, bream, deep learning, methylation detection, qitan

## Abstract

Nanopore sequencing enables the simultaneous detection of genetic sequences and DNA modifications, yet the development of accurate, open-source computational models for these tasks, particularly for non-ONT platforms, remains challenging. To address this, we developed Bream, an open-source deep learning framework that integrates a convolutional neural network with a reverse long short-term memory network for base calling and a bidirectional LSTM with an attention mechanism for methylation detection. We trained and evaluated Bream on datasets from *A. thaliana, O. sativa*, and *D. melanogaster* generated using a novel nanopore sequencing platform (Qitan Technology’s QCell-384) featuring engineered helicase and nanopore proteins. The framework achieved base-calling accuracies between 89.38% and 91.83%, comparable to ONT’s R9.4 platform, and demonstrated high-performance methylation detection, with an AUC-ROC of 0.98 on a *D. melanogaster* dataset. Furthermore, its estimates of whole-genome CpG methylation frequency showed strong agreement (Pearson’s r ≥ 0.96) with bisulfite sequencing data across species. These results demonstrate Bream as a powerful, transparent, and adaptable tool that facilitates simultaneous base calling and methylation detection on emerging nanopore sequencing platforms, thereby advancing open innovation in the field.

## Introduction

Over the past decade, Oxford Nanopore Technologies (ONT) has introduced a cutting-edge nanopore sequencing platform renowned for its long read lengths, real-time detection of DNA modifications, and portability. These features make it valuable for applications such as complex genome assembly, rapid pathogen detection, and real-time environmental monitoring (Wang et al., 2021). In recent years, additional companies have launched novel nanopore sequencing platforms that aim to lower research costs, broaden application possibilities, and enhance sequencing accuracy. However, the development workflow for these platforms—spanning protein engineering (e.g., helicases, nanopores) and computational algorithms for base calling and modification detection—remains largely inaccessible to many researchers.

Nanopore sequencing operates by detecting changes in electrical resistance as single-stranded DNA or RNA molecules pass through nanopores. These changes generate electrical signals that carry both sequence and modification information. The quality of these signals is influenced by two key factors: 1) the helicase enzyme, which controls the speed of DNA movement through the pore, and 2) the nanopore protein, which determines the electrical signal patterns associated with the five consecutive nucleotides within the pore (Loman et al., 2015). While slower DNA translocation reduces signal noise, it also lowers sequencing throughput. The variability in electrical signals across different nucleotide contexts affects the accuracy of base calling and modification detection. To optimize both throughput and precision, commercial nanopore chips rely on the characteristics of helicase and nanopore proteins. ONT has made continuous improvements to both the helicase and nanopore protein structures, enhancing the sequencing accuracy for base sequences and modifications. In 2018, ONT released the R9.4 sequencing chip, which achieved an average sequencing accuracy of 90% and a throughput of approximately 120 GB per flow cell. In 2022, ONT introduced the R10.4 chip, which improved the performance of both the helicase and nanopore protein, achieving an average sequencing accuracy of 97% while maintaining the same throughput. This advancement has led to even broader adoption of ONT technology. However, the details of the improvements in the helicase and nanopore protein design remain undisclosed.

Another critical challenge in nanopore sequencing platform development is the creation of accurate base calling algorithms, and, subsequently, methylation detection algorithms based on the base calling results. Base calling from nanopore electrical signals suffers from high noise, including interference from base modifications. Over the years, ONT has improved its base calling algorithms, initially using Markov statistical models (David et al., 2017) and later implementing various deep learning architectures (Boža et al., 2017), (Teng et al., 2018), (Zeng et al., 2020), (Huang et al., 2022), (Xu et al., 2021) for signal interpretation. This has resulted in the development of several base-calling software tools, including MinKNOW, Guppy, Bonito (Nanoporetech, 2024), and Dorado (Dorado, 2024). While MinKNOW, Guppy, and Bonito primarily focused on base recognition, Dorado can detect sequences and base modifications simultaneously. Bonito, as a training framework for base recognition, along with the C++-based Dorado version, has enhanced sequencing data processing capacity and increased the sensitivity for detecting DNA methylation. Concurrently, DNA methylation detection capabilities have progressively improved alongside iterations of sequencing chips and base calling algorithms. Tools such as DeepSignal (Ni et al., 2019), DeepSignal-plant (Ni et al., 2021), and DeepMod (Liu et al., 2019), developed for the R9.4 chip and Guppy software, evolved into DeepMod2 (Ahsan et al., 2024), DeepBAM (Bai et al., 2024), and DeepPlant (Chen et al., 2025) for the R10.4 version, demonstrating enhancements in algorithm performance, software capability, and the diversity of detectable methylation types. While these tools, particularly Dorado, enable simultaneous base and modification calling, they are proprietary and tightly integrated with ONT’s proprietary sequencing chemistry.

China is still in the early stages of research and application in nanopore sequencing, with considerable growth potential. As Chinese companies like Qitan Technology continue to rise in the nanopore sequencing area, China is progressively addressing the gaps in areas such as helicase and nanopore protein design, as well as base calling methods. While companies like ONT have partially open-sourced their software and algorithms, the core decoding algorithms of the base calling models remain proprietary. This lack of transparency continues to limit the open development of novel nanopore sequencing techniques.

Here, we develop Bream, an open-source framework that allows simultaneous base calling and methylation detection using raw signals from nanopore-type sequencing platforms. As an example, we report commercial-level helicase and nanopore proteins that have been applied in Qitan Technology’s QCell-384 sequencing chip and obtain raw signals from it for training and testing Bream. The results show that Bream achieves high base calling accuracies and methylation frequency correlations with bisulfite sequencing (BS-seq) across datasets. Our work provides a transparent, trainable tool that advances the open development of computational methods for novel sequencing technologies. Our efforts will facilitate further advancement of nanopore sequencing technology.

## Results

### Protein screening and statistics of raw sequencing signals for a novel nanopore sequencing platform

**FIGURE 1:**
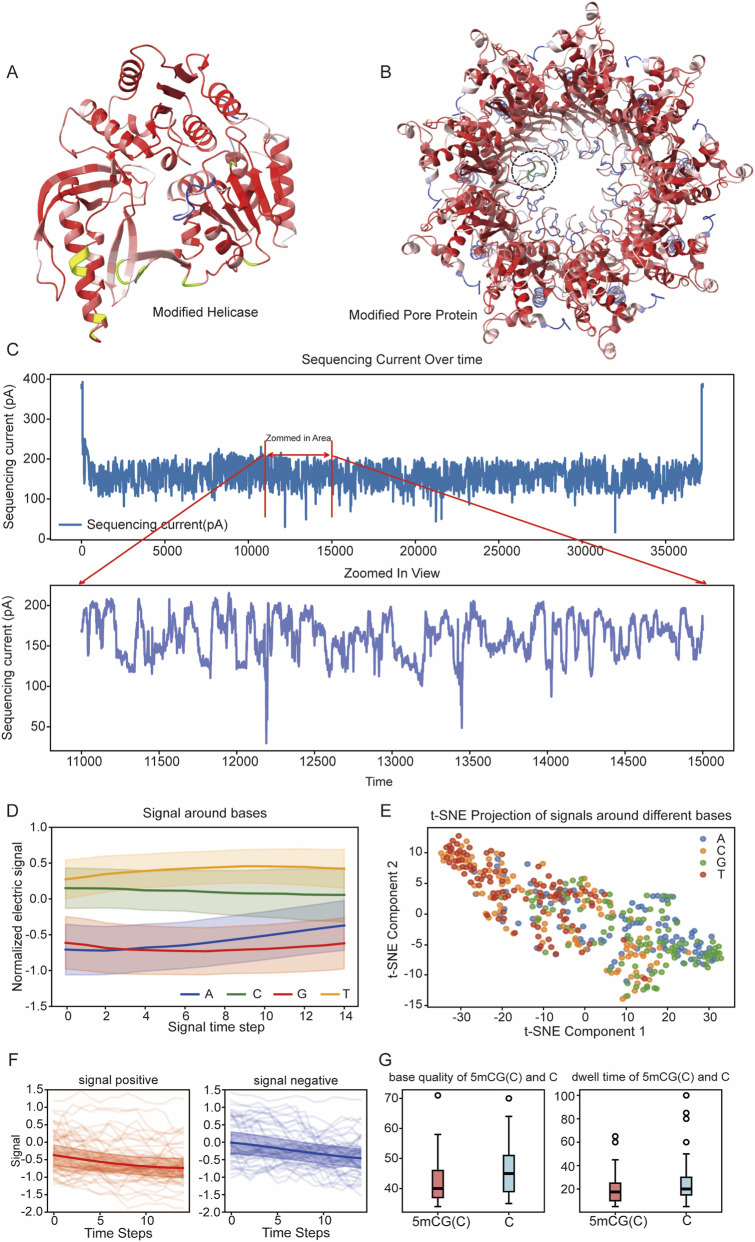
Analysis of Qitan Nanopore Sequencing Signals and Model Outputs for Different Bases and Methylation Status. **(A)** Structure of the modified Pif1-like helicase with cysteine/unnatural amino acid incorporation. The green region highlights the mutated area. **(B)** Mutated pore protein from the CsgG/HfaB family of *Pseudomonas* species. The circled region indicates the mutated area. **(C)** Sequencing current over time, the upper part of the figure shows a complete raw read signal, while the lower part shows an enlarged area of the read. This figure demonstrates the random fluctuations and instability of the signal. **(D)** Normalized electric signals around nucleotide bases (A, C, G, T), the signals around bases were located by the output of basecalling model. The shaded area represents the standard deviation range of these signals. **(E)** t-SNE projection of sequencing signals corresponding to different bases. **(F)** Temporal signal patterns for randomly selected 1,000 positive and negative signals. **(G)** Comparison of base quality scores and dwell times for methylated (5mCG(C)) and non-methylated **(C)** bases, showing minor differences.

To mitigate the challenge of rapid polynucleotide translocation through nanopores that complicates signal discrimination, we engineered a Pif1-like helicase through random amino acid mutations. Specifically, cysteine residues and unnatural amino acids were introduced into critical structural domains, including the tower (residues E264-P278, N296-A394), pin (K89-E105), and 1A domains (M1-L88, M106-V181) (Figure 1A). These modifications enhanced helicase-DNA binding and stabilized translocation at 400 bp/s, a rate comparable to ONT R9.4 flow cells (450 bp/s) and superior to earlier R9 iterations (250 bp/s) (Wang et al., 2021). The engineered helicase thus effectively regulates nucleic acid movement, improving the signal-to-noise ratio in downstream measurements.

In parallel, we redesigned the nanopore protein, derived from the CsgG/HfaB family of *Pseudomonas* species, by mutating amino acids 69–76 (KPTPASSF) to RPSPASAQ (Figure 1B). This variant exhibited improved structural rigidity and stronger nucleic acid affinity, leading to enhanced electrical signal stability. Together, the modified helicase and pore enabled sequencing with base calling accuracies approaching 90% and methylation detection correlations ≥96% with bisulfite sequencing (BS-seq) (see in following sections), comparable to ONT’s R9.4 platform (Wang et al., 2021).

To evaluate signal characteristics, we analyzed representative raw electrical traces from sequencing reads (Figure 1C). Signals outside the DNA-bound state exhibited higher current levels, while DNA-associated signals fluctuated within the 100–200 pA range. Zoomed-in views revealed sporadic drops, indicative of local signal instability—suggesting room for further pore optimization.

Next, we computed the average signal values across 100 intervals of 15-base segments for each canonical base (A, C, G, and T), and visualized the resulting distributions (Figure 1D). To explore the underlying structure of these signal patterns, we applied t-SNE dimensionality reduction (van der Maaten and Hinton, 2008) to over 400 normalized current signal data points. As shown in Figure 1E, signals associated with different bases formed partially separable clusters, indicating that some base-specific features were preserved. However, due to substantial signal noise and overlap among distributions, traditional statistical methods proved inadequate for robust base identification.

We extended this analysis to methylation-related signals by comparing CpG fully methylated (YF6418; all cytosines in CpGs as 5 mC) and fully unmethylated (YF6419) datasets from *D. melanogaster*. We assessed normalized current signals, average base quality scores, and dwell times at CpG motifs (Figures 1F,G). Across all three metrics, clear differences were observed between methylated and unmethylated samples—most notably in current signal profiles. Despite these distinctions, the noisy nature of the data again hindered the effectiveness of conventional statistical techniques in drawing reliable boundaries between methylated and unmethylated states. These limitations strongly motivated the adoption of deep learning-based models for simultaneous base calling and methylation detection.

### Overview of the bream framework

**FIGURE 2:**
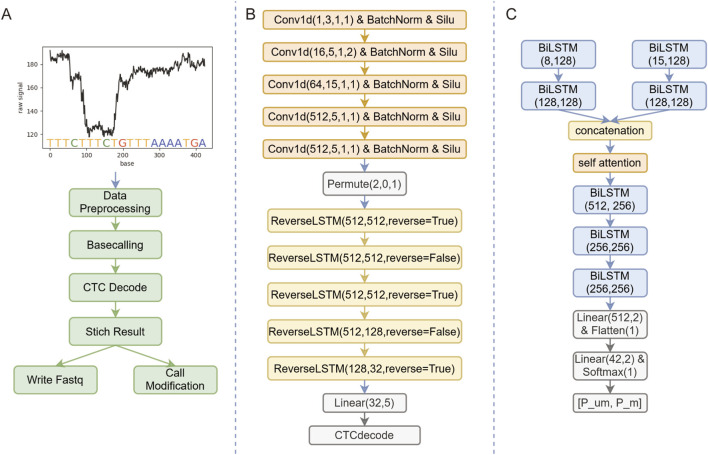
Bream Framework and Model Architectures. **(A)** Workflow of the Bream framework, including signal preprocessing. Basecalling, CTC decoding, result stitching, Fastq writing, and methylation detection. **(B)** Basecalling module architecture, incorporating a five-layer CNN for feature extraction and a five-layer reverse LSTM for temporal pattern recognition. A linear layer followed by a CTC decoder map signals to base sequences. **(C)** Methylation detection module, utilizing BiLSTM networks and attention mechanism to process sequence and signal features, then output probabilities of methylated and unmethylated CpG states after two linear layers.

We introduce Bream, an open-source deep learning framework that enables simultaneous base calling and DNA methylation detection. As depicted in Figure 2A, Bream integrates a comprehensive workflow, processing raw signals to output both sequence data and methylation status. The framework’s core consists of two primary components: a base calling model based on a convolutional neural network (CNN) combined with a reverse long short-term memory (LSTM) network (Figure 2B), and a methylation detection model leveraging a bidirectional LSTM (BiLSTM) with an attention mechanism (Bahdanau et al., 2016) (Figure 2C). Bream achieves high accuracy in decoding base sequences and identifying CpG site methylation directly from nanopore raw signals.

This design ensures a seamless pipeline from input to output, emphasizing clarity and efficiency in handling complex genomic data. By streamlining these processes, Bream advances the capability for integrated analysis in bioinformatics applications.

### Evaluation of base calling using bream on the novel nanopore sequencing platform

We systematically evaluated the performance of the Bream framework using datasets from multiple species, including *A*. *thaliana*, *O*. *sativa*, and *D. melanogaster* with defined methylation states (methylated and unmethylated, treated with methyltransferase (Abdelraheem et al., 2022)). The Bream model, trained on these datasets, achieved efficient base calling across all test datasets (Supplementary Table 5). We then analyzed the quality of the sequencing data and the accuracy of the base calling.

**FIGURE 3:**
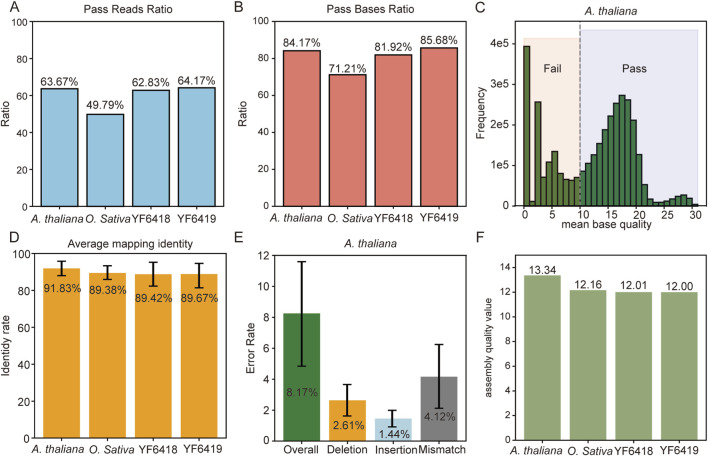
Evaluation of Qitan Sequencing Data Quality and Bream Performance Across Different Datasets. **(A,B)** Pass rates of reads **(A)** and bases **(B)** for *A. thaliana*, *O. sativa*, and synthetic methylated (YF6418) and unmethylated (YF6419) *D. melanogaster* datasets. *A. thaliana* showed the highest pass rates, indicating superior quality. The above results were all counted with a threshold 10 of mean base quality per read. **(C)** Distribution of mean base quality for Arabidopsis reads, highlighting a significant portion of low-quality reads(average base quality ≤ 10). **(D)** Average mapping identity for each dataset, with *A. thaliana* achieving the highest alignment accuracy (91.83%). **(E)** Breakdown of overall, deletion, insertion, and mismatch error rates for *A. thaliana*, showing an overall error rate of 8.19%. **(F)** Assembly quality values for each dataset using Merqury, demonstrating a correlation between alignment quality and assembly quality. **(D–F)** are estimated on reads with mean base quality ≥ 10.

In assessing dataset quality, we calculated the average base quality and evaluated read and base pass rates within the Fastq files of each dataset. Results, depicted in Figures 3A,B, highlight *A. thaliana* as the highest quality dataset, with a read pass rate of 63.67% and a base pass rate of 84.17%. In contrast, *O*. *sativa* demonstrated inferior quality, evidenced by a read pass rate of 49.79% and a base pass rate of 71.21%. Figure 3C further illustrates the quality distribution of *A. thaliana* reads, revealing a notable proportion of low-quality reads characterized by an average quality score ranging from 0 to 2. To evaluate base calling error rates, we employed minimap2 (Li, 2022) to align reads (with an average base quality ≥10) to reference genomes, thereby extracting alignment accuracy (identity) from the CIGAR information. As depicted in Figures 3A,D *thaliana* achieved the highest alignment accuracy at 91.83%, while the YF6418 sample recorded the lowest alignment accuracy at 89.38%. The error, deletion, insertion, and mismatch rates for *A. thaliana* are detailed in Figure 3E, with comprehensive results from the full dataset provided in Supplementary Table 1, indicating a nanopore sequencing error rate ranging from 8% to 10%.

Finally, the accuracy of the assembled sequences derived from base calling was assessed using the software Merqury (Rhie et al., 2020), which evaluates phred base quality values. As illustrated in Figure 3F, there is a correlation between assembly accuracy and alignment accuracy across the various datasets.

### Evaluation of the bream methylation calling model

**FIGURE 4:**
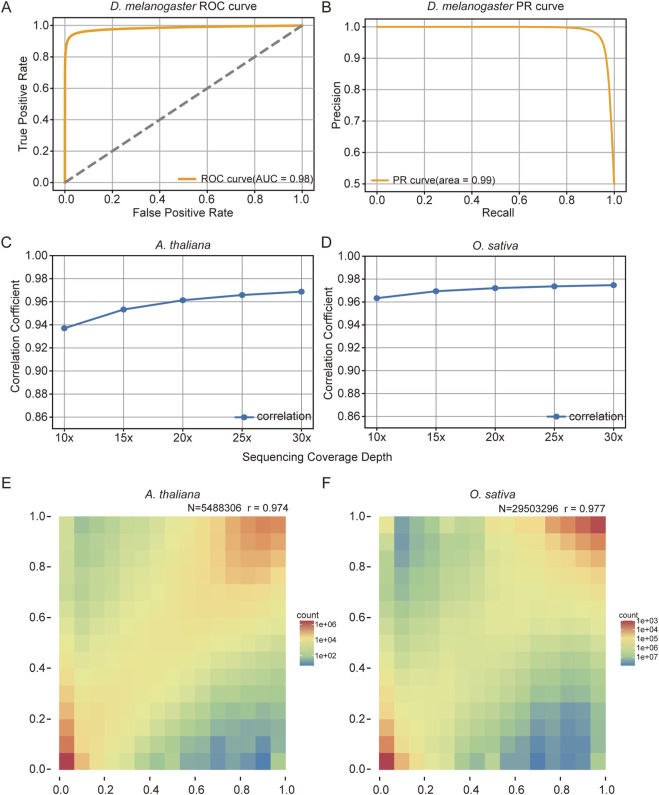
Performances Evaluation of the Bream Methylation Model **(A,B)** Receiver Operating Characteristic (ROC, **(A)** and Precision Recall (PR, **(B)** curves for the *D. melanogaster* dataset, with areas under the curve of 0.98 and 0.99. **(C,D)** Correlation between Bream methylation predictions and whole genome bisulfite sequencing (BS-seq) methylation frequencies for *A. thaliana* **(C)** and *O. sativa* **(D)** at different sequencing depths, showing increasing correlation with higher coverage. **(E,F)** Heatmaps illustrating the consistency between Bream and BS-seq methylation frequencies in *A. thaliana* **(E)** and *O. sativa* **(F)** datasets, with Pearson’s correlation coefficients of 0.974 and 0.977, respectively, indicating strong agreement between the methods.

We trained methylation calling model on *D*. *melanogaster* dataset. Evaluation on the *D*. *melanogaster* dataset included calculating key metrics such as ROC and PR curves (Figures 4A,B), yielding areas under the curve of 98.35% and 98.79%, respectively. Supplementary Table 3 details the model’s precision, recall, and F1 scores at 96.79%, 93.88%, and 95.31%, respectively.

For further validation of real-world data, we analyzed DNA samples from *A. thaliana*, and *O. sativa* leaves using bisulfite sequencing data and nanopore sequencing data. CpG sites with 100% or 0% methylation frequency in the bisulfite data were used as ground truth labels for binary classification. Performance metrics showed that for *A. thaliana*, accuracy, recall, and F1 scores were 94.81%, 92.74%, and 94.70% (Supplementary Table 3), respectively. For *Oryza*, these metrics were 97.38%, 97.33%, and 97.38% (Supplementary Table 3). These results demonstrate that the Bream methylation model has a strong capability for single-molecule CpG methylation detection.

At the genomic level, Bream’s methylation predictions were compared with whole genome BS-seq methylation frequencies by analyzing their correlation across sequencing depths. As shown in Figures 4C,D, the quantitative correlation improved with increasing sequencing depth but plateaued beyond 15× coverage. Heatmaps of *A. thaliana* and *O. sativa* datasets (Figures 4E,F) revealed high consistency between Bream predictions and BS-seq data. In the *A. thaliana* dataset (N = 5,488,306), Pearson’s correlation coefficient was 0.974, while for *O. sativa* (N = 29,503,269), it was 0.977. These results highlight the substantial agreement between Bream and BS-seq in methylation detection while demonstrating Bream’s ability to cover more CpG sites, underscoring its significant advantages in methylation analysis.

### Interference of nanopore sequencing noises on base calling and methylation detection

The accuracy of base calling in nanopore sequencing is highly dependent on signal quality. The technology is susceptible to high levels of noise, and certain sequencing regions may exhibit poor signal characteristics. These high-noise regions, characterized by elevated signal fluctuations, adversely affect read accuracy and the reliability of methylation detection. Specifically, in base calling, high-error regions indicate inherently unreliable reads, which directly compromise downstream analyses such as genome assembly and variant detection. For methylation detection, which relies on discerning subtle signal patterns to identify modified bases, high-error regions lead to misalignment of methylation sites. This introduces inaccuracies in training data, ultimately impairing prediction performance. To mitigate these issues, computational strategies such as error correction or filtering of low-quality regions have been developed. For example, NECAT (Chen et al., 2021) employs an adaptive algorithm to correct errors in *de novo* assembly, while DeepSignal-plant (Ni et al., 2021) refines modification calls by aligning sequences to a reference genome to rectify erroneous k-mers. Nevertheless, the most critical factors for improvement remain continual advancements in sequencing chemistry, flow cell design, and the precision of base calling and methylation detection algorithms.

**FIGURE 5:**
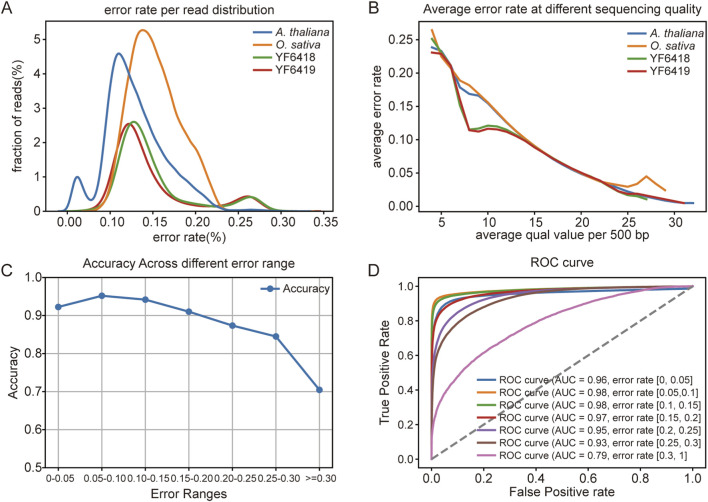
Error Rate Analysis and Methylation Prediction Performance Across Different Datasets **(A)** Distribution of error rates per read across different datasets, showing variability in sequencing accuracy, with *A. thaliana* having the lowest error rates. **(B)** Relationship between sequencing quality and average error rate, indicating a significant decline in error rates with increasing quality values, especially beyond a quality score of 25. **(C)** Methylation prediction accuracy across different error rate ranges, with high accuracy below an error rate of 0.1, and a sharp decline above 0.25. **(D)** ROC curves for methylation prediction under varying error rates, showing a decrease in performance (AUC values) as error rates increase, with the lowest accuracy at error rates ≥0.3.

To evaluate the data quality from our Bream output, we filtered out reads with an average sequencing quality score below 10 across four datasets. We then examined the distribution of average read errors after filtering (Figure 5A). The results show that base calling errors are generally distributed between 0.05 and 0.15, with noticeable variation across different samples. The error distribution for *A. thaliana* is skewed to the left, indicating lower error rates, whereas the error rates for the synthetic *D. melanogaster* datasets (YF6418 and YF6419) are similar. Notably, YF6418, treated with methyltransferase, shows a slight right skew, suggesting that methylation may affect sequencing accuracy.

Furthermore, we explored the relationship between Bream-reported base quality scores and the read accuracy estimated using consensus sequences of corresponding Pacbio HiFi data. Using a 500 bp window, we compared average sequencing quality scores with error rates after aligning the reads to the reference genome (Figure 5B). The result illustrates that as the quality score increases, the error rate decreases significantly. When the average quality exceeds 25, error rates approach zero, indicating a potential correlation between higher sequencing quality and fewer errors.

To evaluate the impact of regions with high signal noise on methylation detection, we analyzed the methylation prediction accuracy under different error rates in the *D. melanogaster in vitro* synthetic datasets (Figure 5C). The results reveal that low error rates (<0.1) lead to high prediction accuracy, while error rates above 0.25 cause a significant drop in accuracy to around 70%. This underscores the importance of maintaining low error rates to ensure reliable methylation detection. Excluding regions with high error rates may improve prediction accuracy by minimizing the impact of poor-quality data.

Finally, we plotted ROC curves and calculated AUC values for methylation prediction at different error rate ranges (Figure 5D). At low error rates (e.g., 0–0.05 or 0.05–0.1), the AUC values approach 1, indicating excellent prediction accuracy. As error rates increase, the ROC curves shift downward, and AUC values decrease. Notably, at error rates ≥0.3, the AUC drops to 0.79, further emphasizing that higher error rates significantly reduce the accuracy of methylation prediction.

## Discussion

This study introduces Bream, a deep learning-based open-source framework for simultaneous base calling and DNA methylation detection on novel nanopore sequencing platforms. Our evaluations demonstrate that Bream achieves robust performance, with alignment error rates consistently below or near 10%, and CpG methylation detection achieving Pearson correlation coefficients ≥0.96 with bisulfite sequencing (BS-seq) across diverse species. These findings underscore the reliability and versatility of Bream in processing noisy electrical signals from emerging nanopore technologies and in capturing both genetic and epigenetic information.

Bream addresses a critical gap in the current nanopore sequencing ecosystem. Existing tools from Oxford Nanopore Technologies (ONT), such as Guppy and Dorado, have evolved from statistical models like hidden Markov models to complex deep learning architectures that support methylation calling (Boža et al., 2017; Teng et al., 2018; Zeng et al., 2020; Huang et al., 2022; Xu et al., 2021; Nanoporetech, 2024; Dorado, 2024). However, the underlying models, training data, and decision mechanisms remain proprietary, limiting reproducibility and independent benchmarking. Academic research has typically been constrained to either (i) base calling using ONT-trained models (Boža et al., 2017) (Teng et al., 2018), (Zeng et al., 2020) (Huang et al., 2022) (Xu et al., 2021) or (ii) methylation detection using signal-level features derived from reads basecalled by Guppy or Dorado (Bai et al., 2024) (Chen et al., 2025). In contrast, Bream is designed to be transparent, trainable, and adaptable, facilitating end-to-end development of base calling and methylation detection pipelines.

Beyond filling a methodological gap, the significance of Bream lies in its potential to democratize and accelerate innovation in nanopore sequencing. As research groups and biotech companies increasingly seek alternatives to proprietary systems, open-source frameworks like Bream can serve as a foundation for customizing base calling models to specific experimental conditions, novel pore chemistries, or species of interest. This is particularly relevant in light of efforts to improve detection of non-CpG methylation (e.g., CHG, CHH (Chen et al., 2025)), RNA modifications (e.g., m6A (Wu et al., 2024)), or even DNA adducts induced by chemical damage (Reverdatto et al., 2022), which require retrainable, modular architectures.

Although Bream demonstrates performance comparable to ONT R9.4, our study highlights persistent challenges. The current signal quality from Qitan Technology’s nanopore sequencing platform is limited by biochemical and engineering factors, including the design and function of helicase and nanopore proteins. As signal-to-noise ratios and dwell-time consistency are crucial for accurate sequence inference, further protein engineering-through rational design or AI-driven sequence optimization-will be essential to enhance signal resolution (Jiang et al., 2025).

On the computational side, the integration of transformer-based architectures-which have shown promise in speech recognition and genomics-could further boost performance by modeling long-range dependencies in signal traces more effectively than RNNs or LSTMs alone (Zhang et al., 2021) (Li et al., 2024). Moreover, extending Bream to detect multiple modification types (e.g., 5hmC, 6 mA) in a multi-task setting would greatly expand its utility for epigenomic studies.

In conclusion, Bream represents a substantial advance in the development of open-source tools for nanopore sequencing. By enabling simultaneous base calling and methylation detection, it sets a new standard for flexibility, transparency, and performance in long-read sequencing analysis. Continued improvements in both hardware (through refined protein design) and software (through model architecture innovation) will be critical for realizing the full potential of nanopore-based genomics. As such, Bream offers a valuable resource to the scientific community and a springboard for further breakthroughs in portable, real-time sequencing technologies.

## Methods

### Expression and purification of recombinant pifi-like helicase and mutant pore proteins in BL21 (DE3) cells

Recombinant plasmids containing sequences of either Pifi-like helicase or mutated pore proteins (amino acid sequences detailed in Supplementary Table 7) were transformed into BL21 (DE3) competent cells using a heat-shock protocol. Following transformation, the cells were plated on solid LB agar containing ampicillin and incubated overnight at 37 °C. Single colonies were selected and cultured in liquid LB medium supplemented with ampicillin, shaken at 200–220 rpm at 37 °C. Optical density (OD600) was periodically measured to monitor cell growth.

Protein expression was induced upon reaching specific optical densities: OD600 of 0.6–0.8 for helicase cultures and OD600 of 2.0–2.2 for pore protein cultures. The cultures were cooled to 16 °C–18 °C, and IPTG was added at final concentrations of 1 mM for helicase or 0.015 mM for pore proteins. Induction was maintained for 12–24 h.

Post-induction, cells were harvested via centrifugation, lysed by high-pressure homogenization, and the target proteins were purified. Pifi-like helicase was purified using FPLC, while pore proteins were isolated through Ni-NTA affinity chromatography. Eluted protein samples were collected for further analysis.

### Base calling data extraction

We generated initial DNA sequences using the base calling model provided by Qitan Technology. These sequences were aligned to the original electrical signals, and corrections were made using the Minimap2 tool to improve accuracy. During preprocessing, we first removed invalid signals representing the stage before DNA entered the nanopore using signal detection methods. Next, the signals were normalized using the Median Absolute Deviation (MAD) (Ni et al., 2019) (Bai et al., 2024) approach. The data was then divided into segments of 6,000 base pairs (bp) with a 500 bp overlap. Chunks with coverage less than or equal to 95% were filtered out to ensure high-quality input data for base calling model training.

### Overview of the bream framework

The Bream framework comprises six computational modules (Figure 2A): signal preprocessing, basecalling, CTC (Graves et al., 2023) decoding, consensus assembly, FASTQ quality value conversion and writing, and methylation detection. To ensure consistent basecalling, the preprocessing module divides raw signals of varying lengths into overlapping segments, each containing 6,000 sample points. These segments are then processed by the basecalling module to identify nucleotide signals. The CTC decoding module interprets these signals to reconstruct the base sequence. Subsequently, the consensus assembly module integrates overlapping segments from the same read to reconstruct the full sequence along with its corresponding quality scores. The writing module formats this information into a standard FASTQ file. Simultaneously, the methylation detection module analyzes CpG motifs within the reconstructed sequences and reports their methylation status.

### Base calling model architecture and training

The base calling module of Bream (Figure 2B) employs a five-layer convolutional neural network (CNN) followed by a five-layer bidirectional long short-term memory (BiLSTM) network. Input signal segments are processed through convolutional layers and batch normalization to extract local signal features and standardize variations. The resulting features are then passed to the bidirectional LSTM layers, which capture contextual information from both forward and reverse directions. A linear layer subsequently converts these features into probabilities for each of the four nucleotide bases (A, C, G, T) or a blank symbol at every position. Finally, a connectionist temporal classification (CTC) decoder translates the probability sequence into the final base sequence. The CTC approach enables unsupervised alignment by allowing variable-length output sequences without strict signal-to-base correspondence, while probabilistically merging paths to resolve input-output length discrepancies.

The training dataset comprised synthetic data from *A*. *thaliana*, *O. sativa*, and *D*. *melanogaster* (datasets YF6418 and YF6419), with a sampling ratio of 4:2:2:2. Approximately 5 million signal segments were extracted for training. Training was conducted on a server equipped with an AMD 9654 CPU and two NVIDIA L40s GPUs. A batch size of 256 was used with the AdamW (Loshchilov and Hutter, 2019) optimizer, an initial learning rate of 0.001, and a linear warm-up phase followed by cosine decay scheduling. To accelerate training, PyTorch’s automatic mixed precision was employed. The entire training process spanned approximately 80 epochs over 3 days. During inference, the model was converted to half-precision for GPU execution, significantly improving throughput (measured in bases called per second).

### Methylation model architecture and training

The methylation detection module (Figure 2C) employs a bidirectional long short-term memory network augmented with an attention mechanism. This module integrates two types of features: (1) sequence-based features, represented as one-hot encodings of the 21-base window centered on each CpG site, and (2) normalized raw signal features derived from the corresponding genomic region. These features are processed independently through separate BiLSTM layers to capture methylation-related patterns, after which their outputs are concatenated. An attention layer dynamically weights the contributions of these features, followed by additional BiLSTM and linear layers that compute probabilities for unmethylated (*P*_um_) and methylated (*P*_m_) states at each CpG site.

The Bream methylation model was trained with fully methylated (CpG methyltransferase-treated) and fully-unmethylated (PCR-treated) *D. melanogaster* datasets. These annotated datasets were balanced with an equal ratio of positive and negative samples (1:1), randomly partitioned into 90% for training and validation, and 10% for testing model performance. To train the model, methylation-related features were extracted using basecall sequences generated by the Bream model and alignment data. The model was trained on approximately 100 GB of synthetic data from *D*. *melanogaster*, optimized with the Adam optimizer (Kingma and Ba, 2017) at a learning rate of 0.001 and a linear decay factor of 0.4.

### Evaluation of base calling and methylation detection

To assess dataset quality and model performance, we systematically evaluated base calling results. Only reads with an average base quality value (Q-score) ≥10 were retained for downstream analysis, a threshold selected based on previous studies (Zhang et al., 2021) and the overall performance of Bream on the Qitan platform. Raw data quality was evaluated by calculating the average base quality, read pass rate, and base pass rate from the FASTQ files of each dataset. To assess base calling error rates, filtered reads were aligned to the reference genome using minimap2. Alignment accuracy (identity) along with error, deletion, insertion, and mismatch rates were derived from the CIGAR information. Finally, the accuracy of the assembled sequences generated through base calling was evaluated using Merqury.

For methylation data, in addition to presenting classification accuracy using ROC curves on a methyltransferase-treated *Drosophila* dataset, we calculated the Pearson correlation coefficient between per-site methylation frequencies derived from our method and those obtained from second-generation bisulfite sequencing in Arabidopsis and rice. The coefficient is defined as:

$$\rho_{X,Y}=\frac{Cov\left(X,Y\right)}{\sigma_{X}\sigma_{Y}}$$

Where Cov denotes covariance, and $\sigma_{X}$ and $\sigma_{Y}$ represent the standard deviations of data X and Y, which correspond to per-site methylation frequencies calculated by our method and bisulfite sequencing, respectively.

## Data Availability

The original contributions presented in the study are publicly available. This data can be found in the National Genomics Data Center (NGDC)’s Genome Sequence Archive (GSA) at https://ngdc.cncb.ac.cn/search/specific?db=bioproject&q=PRJCA036025, with the accession number PRJCA036025.
